# Whole Genome Sequencing of a *Chlamydia trachomatis* Strain Responsible for a Case of Rectal Lymphogranuloma Venereum in Italy

**DOI:** 10.3390/cimb45030119

**Published:** 2023-02-23

**Authors:** Antonella Marangoni, Stefano Amadesi, Marielle Ezekielle Djusse, Claudio Foschi, Valeria Gaspari, Tiziana Lazzarotto, Paolo Gaibani

**Affiliations:** 1 Section of Microbiology, Department of Medical and Surgical Science (DIMEC), University of Bologna, Via Massarenti 9, 40138 Bologna, Italy; 2 Microbiology Unit, IRCCS Azienda Ospedaliero-Universitaria di Bologna, Via Massarenti 9, 40138 Bologna, Italy; 3 Dermatology Unit, IRCCS S. Orsola-Malpighi Hospital, Via Massarenti 9, 40138 Bologna, Italy

**Keywords:** *Chlamydia trachomatis*, lymphogranuloma venereum, LGV, WGS

## Abstract

Lymphogranuloma venereum (LGV) is a systemic sexually transmitted infection caused by *Chlamydia trachomatis* serovars L1 to L3. The current LGV cases in Europe are mainly characterized by an anorectal syndrome, spreading within men who have sex with men (MSM). Whole-genome sequencing of LGV strains is crucial to the study of bacterial genomic variants and to improve strategies for contact tracing and prevention. In this study, we described the whole genome of a *C. trachomatis* strain (LGV/17) responsible for a case of rectal LGV. LGV/17 strain was isolated in 2017 in Bologna (North of Italy) from a HIV-positive MSM, presenting a symptomatic proctitis. After the propagation in LLC-MK2 cells, the strain underwent whole-genome sequencing by means of two platforms. Sequence type was determined using the tool MLST 2.0, whereas the genovariant was characterized by an *ompA* sequence evaluation. A phylogenetic tree was generated by comparing the LGV/17 sequence with a series of L2 genomes, downloaded from the NCBI website. LGV/17 belonged to sequence type ST44 and to the genovariant L2f. Nine ORFs encoding for polymorphic membrane proteins A-I and eight encoding for glycoproteins Pgp1-8 were detected in the chromosome and in the plasmid, respectively. LGV/17 was closely related to other L2f strains, even in the light of a not-negligible variability. The LGV/17 strain showed a genomic structure similar to reference sequences and was phylogenetically related to isolates from disparate parts of the world, indicative of the long-distance dynamics of transmission.

## 1. Introduction

Lymphogranuloma venereum (LGV) is a systemic sexually transmitted infection (STI) caused by *Chlamydia trachomatis* serovars L1 to L3 [1,2].

Since 2003, several outbreaks of LGV have spread across Europe and other high-income countries, mainly among HIV-positive men who have sex with men (MSM) [3,4]. The current LGV cases are essentially characterized by a proctitis, manifesting with anorectal ulceration, tenesmus, anal pain and mucous or bloody discharge (anorectal syndrome) [5,6]. However, several cases of LGV have recently been detected in asymptomatic subjects (up to 30–40% of cases), as well as in lower-risk groups of MSM (e.g., HIV-negative patients) [7].

The current LGV epidemic in Western Europe is caused by the L2 biovar of *C. trachomatis* with a predominance of the L2b genovariant [8]. Nevertheless, many countries have been reporting increasing cases due to other L2 variants, such as L2f or the hybrid L2-L2b/D-Da [2,9,10,11].

Laboratory diagnosis of LGV is mainly based on nucleic acid amplification techniques (NAAT) able to detect *C. trachomatis* and differentiate LGV from non-LGV serovars (e.g., assays based on *ompA* or *pmpH* genes) [12,13]. Other diagnostic procedures, such as microbial cultures, have a limited use and present critical issues. Indeed, *Chlamydia* cultures are impaired by the obligate intracellular nature of the microorganism, requiring the use of cell lines and trained personnel, incurring high costs and long time [14].

Beyond the technical difficulties, chlamydial culture has a critical role, allowing researchers to obtain the viable bacterial strain for further phenotypic and genotypic studies, such as whole genome sequencing (WGS). In this context, some culture-independent methods have been recently developed to sequence the whole genome of *C. trachomatis* directly from clinical samples [10].

WGS-based studies of chlamydial strains can be useful for different reasons, such as: (i) to deepen virulence and pathogenicity factors; (ii) to discover and monitor the presence of new genomic variants; (iii) to decipher the spread of clonal complexes during outbreaks; (iv) to improve strategies for contact tracing and the prevention of LGV spread; (v) to study LGV genomic evolution over the years in different countries.

Considering that limited WGS data of LGV strains are available, herein we sequenced the whole genome of a *C. trachomatis* strain (LGV/17) responsible for a case of rectal LGV, isolated from a symptomatic MSM in 2017 in the North of Italy.

## 2. Materials and Methods

### 2.1. Strain Isolation and LGV Diagnosis

A *C. trachomatis* strain, named LGV/17, was isolated in May 2017 from a rectal swab submitted to the Microbiology Unit of IRCSS Sant’Orsola-Malpighi Hospital of Bologna (Italy) [15]. This strain was recovered during routine diagnostic procedures from a 37-year-old HIV-positive MSM attending the STI Outpatient Clinic of the hospital and presenting with an anorectal syndrome.

The patient underwent a clinical visit, and a rectal swab was collected in a sucrose-phosphate-glutamine (SPG) medium. A part of the sample was processed by a commercial NAAT (Versant CT/GC DNA 1.0 Assay; Siemens Healthcare Diagnostics, Terrytown, USA), revealing a positive result for *C. trachomatis* [16]. Subsequently, a molecular genotyping, based on an *ompA* gene semi-nested PCR and RFLP analysis, identified a *C. trachomatis* L2 serovar [17]. 

The remaining part of the rectal swab was likewise inoculated in LLC-MK2 cells (ATCC^®^ CCL-7). The presence of *C. trachomatis* was confirmed by the demonstration of chlamydial inclusions by direct immunofluorescence, as previously described [18]. After the isolation, the *C. trachomatis* strain was propagated for ∼1/2 weeks in LLC-MK2 cells (ATCC^®^ CCL-7TM). *C. trachomatis* elementary bodies (EBs) were purified from cell debris and reticulate bodies by Renografin density gradient centrifugation, as described elsewhere [19].

The study was conducted according to the guidelines of the Declaration of Helsinki and approved by the Ethics Committee of Sant‘Orsola-Malpighi Hospital, Bologna (78/2017/U/Tess). Informed consent was obtained from the subject involved in the study.

### 2.2. Whole Genome Sequencing and Subsequent Analyses

Genomic DNA was extracted from *C. trachomatis* EBs using a DNeasy Blood & Tissue Kit (Qiagen, Basel, Switzerland), following the manufacturer’s instructions, and further cleaned up with AMPure XP magnetic beads (Beckman Coulter, Milan, Italy). Whole-genome sequencing was performed using both Illumina iSeq 100 (Illumina, San Diego, CA, USA) and Oxford Nanopore MinION (Oxford Nanopore Technologies, Oxford, UK) systems, as previously described [20]. Briefly, short-read sequences were obtained using the Illumina iSeq 100 platform (iSeq Reagent Kit v2; Illumina, San Diego, CA, USA), using the iSeq Reagent kit v2 with 2 × 150 paired-end reads after Illumina DNA Prep paired-end library preparation. Long-read sequences were obtained by the Oxford Nanopore MinION (Oxford Nanopore Technologies, Oxford, UK) platform using a one-direction (1D) library prepared with a rapid sequencing kit (SQK-RAD004) and sequenced on a MinION Mk1C with SpotON flow cell R9.4 with the stand-alone version of MinKNOW v1.15.2 software with local base-calling.

A read quality evaluation was performed with FastQC 0.11.9 and seqtk 1.3. Prior to genome assembly, a filtering step was conducted to remove any contaminant human reads. First, the reference human genome GRCh38 (available at https://ftp.ncbi.nlm.nih.gov/genomes/all/GCA/000/001/405/GCA_000001405.15_GRCh38/ accessed on 1 November 2022) was downloaded from the NCBI website and indexed with the Burrows–Wheeler Aligner (BWA) 0.7.17 using the index command. Next, Illumina and Nanopore reads were mapped to the reference human genome with default parameters using BWA and Minimap2 2.17, respectively. Last, Samtools 1.6 was used to sort the .sam alignment files with the sort command, extract the unmapped reads from the files using the view command with the -f parameter, and convert the resulting files to a .fastq format. Filtered reads were used for de novo genome assembly with a hybrid approach using Unicycler 0.5.0. Assembly quality was evaluated using Quast 5.0.2 and Bandage 0.8.1.

The genome nucleotide sequence was annotated using RASTtk 2.0, and virulence genes were detected by aligning the genome against the Virulence Factor Database’s (VFDB’s) full protein sequence dataset with diamond 2.0.15. Sequence type was determined using the tool MLST 2.0 (https://cge.food.dtu.dk/services/MLST/ accessed on 5 December 2022) from the Center for Genomic Epidemiology (CGE) website. The plasmid nucleotide sequence was aligned to the NCBI non-redundant (nr) database using blastn 2.12.0 in order to identify potential high-homology plasmids.

ParSNP 1.7.4 was launched to generate a phylogenetic tree based on core genome single nucleotide polymorphisms (cgSNPs). *Chlamydia trachomatis* L2 genomes were downloaded from the NCBI website and used for the phylogenetic analysis. Strain 434/Bu (GenBank accession number GCA_000068585) was used as a reference. The phylogenetic tree was reviewed and manually annotated with metadata from the NCBI database using iTOL v6.

Characterization of LGV genovariants was performed through an evaluation of the *ompA* gene variant, as previously described [2]. Briefly, BLAST analysis was performed by aligning the whole-genome of the LGV/17 strain against the LGV *ompA* gene variants available in GenBank representing the different genovariants (i.e., L2a, L2, L2b, L2c, L2d, L2e, L2, L2g, L2bV1, L2bV2, L2bV3, L2bV4, and L2-L2b/D-Da).

### 2.3. Data Availability

The complete genome sequence of the *C. trachomatis* strain LGV/17 was deposited in the NCBI database and is freely accessible at BioSample, accession number SAMN31133397.

## 3. Results

We generated a high-quality genome assembly of the clinical isolate LGV/17 using Illumina and Nanopore sequencing technologies. Illumina sequencing produced a total of 1,711,892 paired-end reads with an average Phred quality score of 35. Nanopore sequencing generated 102,458 reads ranging from 109 to 69,834 bp, with an average length of 3432 nucleotides and a mean Phred score of 20. After removing reads that mapped to the human genome, a total of 397,163 (23.2%) Illumina reads and 51,862 (50.6%) Nanopore reads were selected for the *C. trachomatis* genome assembly. The resulting de novo assembly produced a complete and circular genome with a total length of 1,045,668 bp, which consisted of a 1,038,168 bp chromosome and a 7500 bp plasmid. The mean coverage was 280×, and the G+C content was 41.3%. The genome was identified as sequence type ST44, and Blast analysis of the *ompA* gene variants showed that it belonged to the genovariant L2f (100% identity and coverage to *ompA* of the reference *C. trachomatis* isolate 128C/07 (EU676181)).

**Figure 1:**
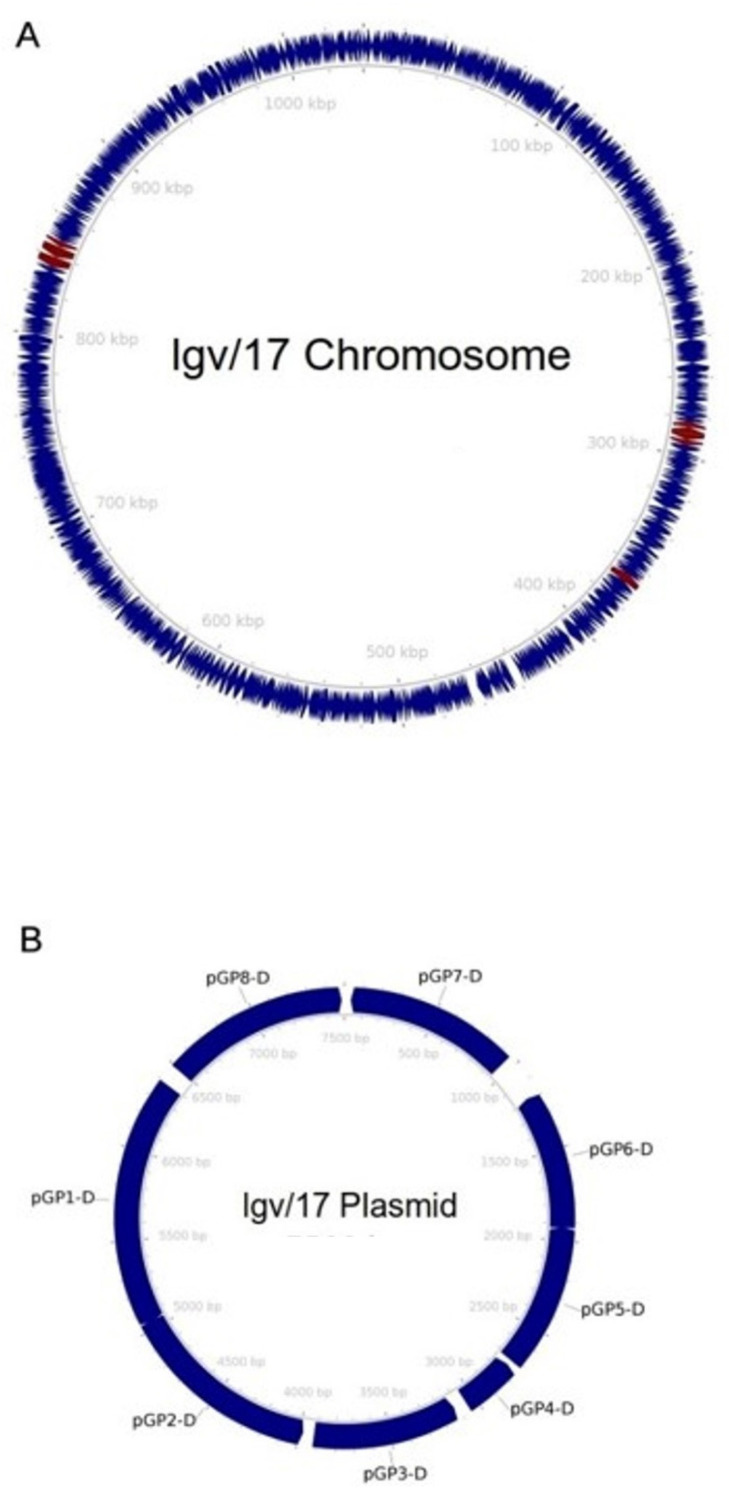
Graphic representation of the LGV/17 genome. The chromosome is shown in panel (**A**), while the plasmid is in panel (**B**). Coding sequences are shown as navy-blue arrows. Maroon arrows indicate genes related to virulence mechanisms.

Functional annotation of the genome identified 953 protein-encoding sequences and 37 tRNAs on the chromosome (Figure 1). As shown in Table 1, we identified nine ORFs involved in virulence mechanisms, clustered in three genomic locations. These encoded for polymorphic membrane proteins (Pmps) A-I, autotransporter adhesins involved in the initial phase of *C. trachomatis* infection [21]. Eight ORFs encoding for glycoproteins Pgp1-8 were also found in the plasmid (Figure 1). The plasmid nucleotide sequence showed high homology to the plasmid described in 1988 by Comanducci et al. and believed to be required for *C. trachomatis* growth within mammalian cells (Query Cover 100%, Identity 99.98%) (GenBank Accession Number: X07547.1) [22].

**Figure 2:**
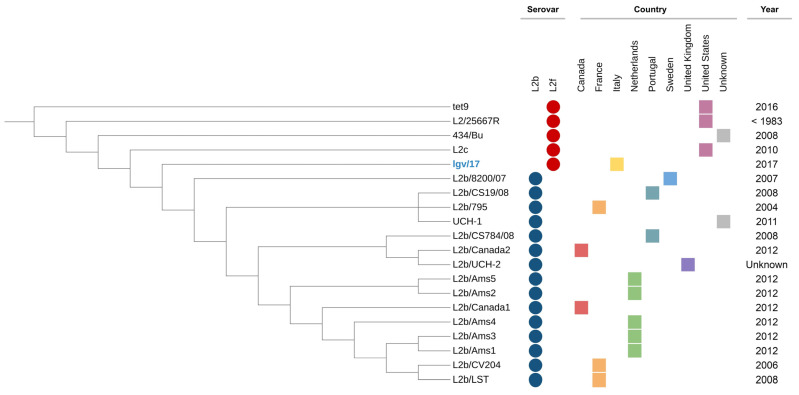
Phylogenetic tree annotated with metadata from each strain. Serovars are indicated by colored circles, while the countries are represented by colored squares.

Phylogenetic analysis (Figure 2) indicated that, even in light of the significant variability, the LGV/17 strain is closely related to the other strains identified over the years as the L2f genovariant in other countries.

## 4. Discussion

To the best of our knowledge, this is the first report about the WGS of an LGV-associated *C. trachomatis* strain isolated from Italy. This work expands the existing literature on *C. trachomatis* genomic data, which could potentially be used to help in detecting, monitoring, and controlling LGV spread and outbreaks. Moreover, these data could be useful for the study of the genomic evolution of LGV over time and across different countries, as well as for deepening the pathogenesis and virulence of *C. trachomatis* biovars.

At first, we found that the LGV/17 strain belonged to L2f genovar. As previously reported [2], L2f was found to be the most common genovar in the urban areas of Bologna (North of Italy) in the years 2016–2017 and 2017–2018, accounting for about 50% of all LGV cases detected during these periods. In agreement with Marangoni et al., we hereby confirm the association between L2f genovar and the presence of rectal signs and symptoms (i.e., tenesmus, rectal discharge, anal pain), as well as other STIs, such as HIV [2].

Multi-locus sequence typing (MLST) has become a useful tool for studying the genetic diversity of important public health pathogens, leading to improvements in the monitoring and molecular epidemiology of *C. trachomatis* infections [23,24]. Several MLST-like systems able to identify LGV strains have been previously proposed, but none of them has been broadly adopted by the scientific community [25,26,27]. In silico extraction of MLST profiles using the Chlamydiales scheme [25], currently available at PubMLST (https://pubmlst.org/ accessed on 5 December 2022), showed that the LGV/17 strain belongs to sequence type 44 (ST44), being indistinguishable from the parent-like L2b/UCH-1/proctitis lineage [10].

Compared to MLST, the WGS approach has a higher discriminatory power, allowing timely characterization of circulating types and variants, which can help to disclose transmission chains, guide therapies, and identify emerging public-health harm [10]. However, WGS-based typing is still not applicable for routine surveillance of *C. trachomatis* because of several limitations, such as costs and technical difficulties. 

By means of a chromosome functional annotation, we identified nine ORFs encoding for the polymorphic membrane proteins (Pmps) A-I, clustered in three genomic locations. Pmps are a group of membrane-bound surface-exposed proteins that have been characterized as autotransporter adhesins, crucial for the initial phases of chlamydial infection [21]. These proteins are differently regulated in response to stress (e.g., beta-lactams) and also play an important role as potent antigenic proteins involved in the immunopathogenesis of chlamydial infections [28]. Interestingly, the expression of Pmps is variably regulated in different chlamydial serovars and may induce differential immune responses with specific serovars [29].

Pmps are transcribed in vitro in various *C. trachomatis* strains including L2 and are all translocated to the bacterial surface [30,31]. However, it has been demonstrated that Pmps exhibit variable transcription patterns: for example, pmpD gene from the *C. trachomatis* serovar L2 upregulated when reticulate bodies (RBs) differentiated into elementary bodies (EBs) (i.e., about 16–24 h post-infection) [31].

When looking at the LGV/17 plasmid, eight ORFs encoding for glycoproteins Pgp1-8 were detected. In *C. trachomatis* serovar L2, the plasmid is known to regulate the expression of more than 20 genes at the transcription level [32,33], and plasmid-free organisms displayed reduced expression levels of glycogen synthesis genes, lacking glycogen accumulation in inclusions [33].

The significant role of plasmid glycoproteins Pgp1-8 has been recognized by different studies [32]. In fact, it has been shown that Pgp1, -2, -6, and -8 are critical for plasmid maintenance, that Pgp4 is a transcriptional regulator of chlamydial gene expression and glycogen synthesis, while Pgp3, -5, and -7 are necessary for chlamydial growth in vitro [34,35].

Finally, we conducted a phylogenetic analysis by comparing the entire LGV/17 sequence with a series of *C. trachomatis* L2 genomes downloaded from the NCBI website. As expected, our strain was closely related to other L2f strains, despite a not-negligible variability. These data potentially indicate a ‘genetic drift’ of L2 strains over the years and in different regions/areas of the world. The city where the LGV/17 strain was isolated is a high-density urban area (i.e., Bologna) with a significant exchange of people coming from different European/non-European countries. Thus, the creation of different sexual networks and dynamics of transmission could lead to a complex epidemiological scenario, where different LGV genovariants co-exist, co-evolve, and spread within the population.

## 5. Conclusions

In conclusion, we described the whole-genome of a L2f *C. trachomatis* strain responsible for a rectal LGV in an Italian HIV-positive MSM. The LGV/17 strain showed a genomic structure similar to reference sequences and it was phylogenetically related to isolates from disparate parts of the world, indicative of long-distance dynamics of transmission. Further perspectives include: (i) the isolation and collection of other LGV strains from Italy, to better understand *C. trachomatis* genomic evolution; (ii) in depth studies of the in vitro behavior of the LGV/17 strain (e.g., interactions with different cellular types).

## Figures and Tables

**Table 1:** List of genes encoding for Pmps. Respective locations on the LGV/17 strain chromosome are shown.

Gene	Description	Location
*pmpA*	polymorphic membrane protein PmpA	845,095–848,022
*pmpB*	polymorphic membrane protein PmpB	839,707–844,956
*pmpC*	polymorphic membrane protein PmpC	834,207–839,531
*pmpD*	polymorphic membrane protein PmpD	364,682–369,274
*pmpE*	polymorphic membrane protein PmpE	291,344–294,241
*pmpF*	polymorphic membrane protein PmpF	288,243–291,341
*pmpG*	polymorphic membrane protein PmpG	285,037–288,078
*pmpH*	polymorphic membrane protein PmpH	281,977–285,006
*pmpI*	polymorphic membrane protein PmpI	278,235–280,871

## Data Availability

The complete genome sequence of *C. trachomatis* strain LGV/17 is freely available at BioSample accession number SAMN31133397.
